# Supplementary material for: Urine C-peptide creatinine ratio can be used to assess insulin resistance and insulin production in people without diabetes: an observational study
Source: BMJ Open. 2013 Dec 18;3(12):e003193. doi: 10.1136/bmjopen-2013-003193 (PMC3884748; doi:10.1136/bmjopen-2013-003193)
Supplement: Author's manuscript [file bmjopen-2013-003193.draft_revisions.pdf]

Urine C-Peptide Creatinine Ratio: A novel method for the assessment of insulin resistance and insulin production in people without diabetes.

Journal:	*BMJ Open*
Manuscript ID:	bmjopen-2013-003193
Article Type:	Research
Date Submitted by the Author:	09-May-2013
Complete List of Authors:	Oram, Richard; University of Exeter, NIHR Exeter Clinical Research facility; Royal Devon and Exeter NHS Foundation Trust,<br>Rawlingson, Andrew; Royal Devon and Exeter NHS Foundation Trust, ; University of Exeter, NIHR Exeter Clinical Research Facility<br>Shields, Beverley; University of Exeter, NIHR Exeter Clinical Research Facility<br>Bingham, Coralie; Royal Devon and Exeter NHS Foundation Trust,<br>McDonald, Timothy; Royal Devon and Exeter NHS Foundation Trust, ; University of Exeter, NIHR Exeter Clinical Research Facility<br>Knight, Bridget; University of Exeter, NIHR Exeter Clinical Research Facility<br>Hattersley, Andrew; University of Exeter, NIHR Exeter Clinical Research Facility
**Primary Subject Heading**:	Diabetes and endocrinology
Secondary Subject Heading:	Epidemiology
Keywords:	Epidemiology < TROPICAL MEDICINE, DIABETES & ENDOCRINOLOGY, STATISTICS & RESEARCH METHODS

SCHOLARONE™
Manuscripts

Urine C-Peptide Creatinine Ratio: A novel method for the assessment of insulin resistance and insulin production in people without diabetes.

*Richard A Oram[1,2], *Andrew Rawlingson[1,2], Beverley M Shields[1], Coralie. Bingham[2], Rachel EJ Besser[1],Tim J McDonald[1,2], Bridget A Knight[1], Andrew T Hattersley[1]

*Joint first authors

1. NIHR Exeter Clinical Research Facility, University of Exeter Medical School, Royal Devon & Exeter NHS Foundation Trust, Barrack Road, Exeter, UK.
2. Royal Devon & Exeter NHS Foundation Trust, Barrack Road, Exeter, UK

Word Count: Abstract 200w, article 2241w

Key words: UCPCR, HOMA, Insulin Resistance, C-peptide/urine, Insulin/secretion

Corresponding Author:

Dr Richard Oram

r.oram@exeter.ac.uk

Tel: +44 1392 406807
Fax: +44 1392 406767

Level 2
NIHR Exeter Clinical Research Facility
University of Exeter Medical School
Barrack Road
Exeter
UK
EX2 5DW

For peer review only - http://bmjopen.bmj.com/site/about/guidelines.xhtml

ARTICLE FOCUS

- Large epidemiology studies often use a serum measurement of fasting insulin and glucose to measure insulin resistance.
- Measurement of Urine C-peptide creatinine ratio (UCPCR) is a non-invasive measure of insulin production that can be posted from home.

KEY MESSAGES

- Urine C-peptide creatinine ratio strongly correlates with serum insulin levels, and HOMA calculated insulin resistance in people without diabetes.
- Epidemiology studies of insulin resistance can now be performed without needing blood testing, using a posted urine sample.

STRENGTHS AND LIMITATIONS

- This study uses both a clinical research facility setting, and samples sent from home to demonstrate that UCPCR can be used in healthy volunteers.
- UCPCR is compared to other epidemiological measures of insulin resistance such as fasting insulin and HOMA.
- UCPCR is not valid in people with Chronic Kidney Disease stages 3-5

For peer review only

For peer review only - http://bmjopen.bmj.com/site/about/guidelines.xhtml

ABSTRACT

OBJECTIVES
Current assessment of insulin resistance(IR) in epidemiology studies relies on blood measurement of C-peptide or insulin. Urine C-peptide creatinine ratio (UCPCR) can be posted from home unaided. It is validated against serum measures of insulin in people with diabetes. We tested whether UCPCR could be a surrogate measure of IR by examining the correlation of UCPCR with serum insulin, C-peptide and HOMA2-IR in subjects without diabetes, and in subjects with Chronic Kidney Disease (CKD).

DESIGN
Observational study

SETTING
Single centre Clinical Research Facility

PARTICIPANTS
37 healthy volunteers and 30 patients with CKD (GFR 15-60) were recruited.

PRIMARY AND SECONDARY ENDPOINTS
Serum insulin, C-peptide and glucose at fasting(0), 30, 60, 90 and 120 minutes were measured during an oral glucose tolerance test (OGTT). Second void fasting UCPCR and 120 minutes post OGTT UCPCR were collected. HOMA2-IR was calculated using fasting insulin and glucose. Associations between UCPCR and serum measures were assessed using Spearman's correlations.

RESULTS
In healthy volunteers, fasting second void UCPCR strongly correlated with serum insulin (r_s=0.69, p<0.0001), C-peptide(r_s=0.73, p<0.0001) and HOMA2-IR (r_s=-0.69, p<0.0001). 120min post OGTT UCPCR correlated strongly with C-peptide and insulin area under the curve. In patients with CKD, UCPCR did not correlate with serum C-peptide, insulin or HOMA2-IR.

CONCLUSION
In subjects with normal renal function, UCPCR may be a simple, practical method for the assessment of IR in epidemiology studies.

For peer review only

BACKGROUND

Insulin resistance has been shown to be a significant predictor for the development of diabetes and for cardiovascular risk[1,2]. Understanding the epidemiology of insulin resistance is important in the identification of patients at risk of type 2 diabetes (T2D) and vascular disease, and for the study of prevention. The optimum individual method to assess insulin physiology uses glucose disposal rate during hyperinsulinaemic-euglycaemic clamp studies[3,4], which require infusions of both insulin and glucose and cannot be used at a population level. Minimal model analysis of glucose and insulin levels during intravenous or oral glucose loading allows assessment without the use of intravenous insulin, but still necessitates multiple blood samples[5]. Fasting assessments of insulin alone, or with measures of glucose have been used as a more simple method to study insulin resistance[6], and have been validated against other more invasive tests[7]. One widely used approach that allows for variation in the fasting glucose is the Homeostasis Model Assessment (HOMA, http://www.dtu.ox.ac.uk/homacalculator/index.php)[8,9] that models fasting serum glucose, and insulin or C-peptide levels to calculate a measure of insulin resistance. HOMA requires that a fasting blood sample is taken and the sample is relatively rapidly spun to avoid protease-mediated degradation. This means an appointment with healthcare or research staff is still required and this is not always readily available for some large epidemiological studies.

An alternative method to blood sampling, which allows samples to be provided without outside assistance, is to measure urinary C-peptide. C-peptide is secreted in equimolar amounts to insulin and is filtered in the kidney, with 5% excreted unchanged in the urine, making urinary measures possible[10]. We have recently demonstrated that C-peptide is measureable, reproducible and stable in urine for up to 72 hours in boric acid preservative (allowing postage from primary care or from home)[11]. Measuring C-peptide as a ratio against creatinine allows the use of a single spot urine sample by accounting for dilution in the same way as protein creatinine ratio. In patients with type 1 diabetes (T1D) and T2D, 2 hour urine C-peptide creatinine ratio (UCPCR) is highly correlated to 90 minute serum C-peptide in the standard Mixed Meal Tolerance Test[12,13]. We have also shown that in patients with T2D and mild chronic kidney disease (CKD), the correlation between serum C-peptide and urine is maintained[14]. As fasting serum insulin or C-peptide alone is a helpful marker of insulin resistance in people without diabetes, it may be that UCPCR could also be used in this manner.

For peer review only - http://bmjopen.bmj.com/site/about/guidelines.xhtml

For peer review only

If UCPCR can be used in people without diabetes this practical method could allow large scale, population based assessment of insulin resistance without needing a blood sample to be taken. We aimed to test whether UCPCR could be used as a surrogate measure of insulin resistance in epidemiological studies by examining the correlation of UCPCR with fasting serum insulin, C-peptide and HOMA2-IR in subjects without diabetes. As a secondary outcome we tested whether stimulated UCPCR could be used as a marker of insulin secretion during an oral glucose tolerance test. We also wanted to see if the correlations were maintained in subjects with chronic kidney disease.

METHODS

Study participants

2 groups were recruited from December 2009 to May 2010:

37 healthy controls (22 female) with normal renal function (eGFR>60ml $min^{-1}m^{-2}$), and normal glucose tolerance[15,16] were recruited from research volunteer databases in Devon.

30 patients (8 female) with normal glucose tolerance and a clinical diagnosis of CKD stage 3 or greater (MDRD eGFR<60ml $min^{-1}m^{-2}$) (www.renal.org/CKDguide/full/UKCKDfull.pdf) were recruited from general nephrology clinics at the Royal Devon and Exeter Hospital. Patients on renal replacement therapy (either dialysis or transplant) were excluded from the study.

Table 1		
	Normal renal function group	CKD group
Total subjects	37	30
Female	22	8
Age (years)	50 (29-67)	65(52-71)
BMI (Kg/m^2)	27.0(23.5-33.0)	26.4(24.1-28.6)
HbA1c (%)	5.7(5.4-6.0)	5.9(5.6-6.1)
Fasting Blood Glucose (mmol/L)	4.8(4.5-5.1)	5.0(4.5-5.3)
Creatinine (mmol/L)	77(66-84)	195(134-231)
MDRD eGFR (ml/min/1.73m^2)	88(76-101)	32(26-46)

Table 1 – Cohort Characteristics. Data are presented as median (interquartile range)

All studies were performed with approval from the South West 2 Research Ethics Committee.

For peer review only - http://bmjopen.bmj.com/site/about/guidelines.xhtml

Clinical sampling

Participants fasted from midnight prior to their visit and emptied their bladder on waking (first-void urine). Demographic data, past medical and drug history were recorded. Baseline fasting blood samples were collected for routine analysis of glucose, HbA1c and renal function. A second urine sample (second-void fasting) was collected immediately prior to OGTT for measurement of UCPCR(UCPCR0).

In a standard OGTT (75 g glucose), blood samples were collected at 30, 60 90 and 120 minutes. A further urine sample was collected for UCPCR analysis at 120 minutes (UCPCR120). Blood samples were immediately centrifuged and separated. Serum and urine samples were initially stored at -20°C then transferred and stored at -80°C within 1 week. Serum samples were subsequently analysed for insulin, C-peptide, and glucose. Urine samples were analysed for C-peptide and creatinine and a urine C-peptide creatinine ratio was calculated.

Biochemical analysis

Urine and serum C-peptide analysis were performed by electro-chemiluminescence immunoassay (Roche Diagnostics E170 C-peptide assay). All urine samples were pre-diluted 1:10 with equine serum albumin (diluent multianalyte, Roche Diagnostics, Mannheim, Germany). Serum insulin analysis was performed by electro-chemiluminescence immunoassay (Roche Diagnostics E170 C-peptide assay). Glucose and creatinine were analysed on the Roche P800 modular platforms. All analysis was performed in the Department of Chemical Pathology, Royal Devon and Exeter Hospital. eGFR was calculated using 4-variable MDRD formula[17].

Data analysis

Serial serum C-peptide, insulin and glucose measurements were used to calculate area under the curve (AUC) for each parameter. Insulin resistance (HOMA2-IR) was derived from fasting glucose and insulin [http://www.dtu.ox.ac.uk/homacalculator/index.php]. Associations between second void UCPCR0 and stimulated UCPCR 120 with serum C-peptide, insulin, HOMA2-IR were assessed using Spearman correlations. Analyses were performed separately for the group with CKD and the group without CKD. Data for UCPCR were non-normally distributed so non-parametric statistical testing was used for analysis

For peer review only - http://bmjopen.bmj.com/site/about/guidelines.xhtml

RESULTS

A summary of the characteristics of the study group is shown in Table 1. 3 subjects with CKD had serum C-peptide samples that were not analysed due to sampling problems, their results have been included in analyses excluding those involving C-peptide values.

Table 2		
	Normal renal function group (n=38)	CKD group (n=30)
Fasting C-Peptide (nmol/L)	0.7(0.5-1.0)	1.2(0.8-1.6)
Fasting Insulin (pmol/L)	8.1(5.0-13.1)	8.8(6.4-12.0)
C-Peptide Area Under Curve(nmol/L)	294(207-405)	457(371-550)
Insulin Area Under Curve (pmol/L)	6180(3641-11994)	7685(5050-9597)
UCPCR0 (nmol/mmol)	1.0(0.6-1.4)	0.914(0.5-1.5)
UCPCR120 (nmol/mmol)	3.8(2.3-7.0)	2.8(0.9-4.0)
HOMA2-IR	1.2 (0.8-1.9)	1.3 (0.9-1.7)

Table 2 – Median (Interquartile range) serum insulin, C-peptide, UCPCR and HOMA-IR

Fasting 2nd void UCPCR strongly correlated with serum insulin, C-peptide and HOMA2-IR in people without chronic kidney disease.

In subjects without renal disease fasting second void UCPCR0 strongly correlated with serum insulin (r_s=0.69, p<0.0001), C-peptide(r_s=0.73, p<0.0001) and HOMA2-IR (r_s=-0.69, p<0.0001). Scatter plots with Spearman's correlations and regression lines are shown in Figure1.

Figure 1

Figure 1 Scatter plots showing fasting second void UCPCR (UCPCR0) was strongly correlated to fasting serum insulin (A) and HOMA2-IR (B) in 37 people

For peer review only - http://bmjopen.bmj.com/site/about/guidelines.xhtml

with normal renal function. Regression line Spearman's r_s correlations shown. *=p<0.0001

Stimulated UCPCR values were correlated with stimulated values of serum insulin and C-peptide, in people without chronic kidney disease.

After an OGTT, UCPCR120 values were higher than UCPCR0 (3.8 v 1.0 nmol/mmol, p<0.0001)(Table 2). UCPCR120 correlated with serum insulin (r_s0.78, p<0.0001) and C-peptide area under the curve (r_s0.8, p<0.0001). Scatter plots with Spearman's correlations and regression lines are shown in Figure 2.

Figure 2

Figure 2 Scatter plots showing 120 minute post OGTT UCPCR (UCPCR120) was strongly correlated to serum C-peptide (A) and insulin (B) area under the curve in 37 people with normal renal function. Regression line Spearman's r_s correlations shown. *=p<0.0001

In patients with CKD, UCPCR does not correlate with serum C-peptide, insulin or HOMA2-IR

In patients with CKD, median fasting (1.2 v 0.7 nmol/L p<0.0001) and stimulated (457 v 294 nmol/L, p<0.0001)serum C-peptide measures were higher than the subjects without CKD, but serum insulin levels were not different (7685 v 6180 , p=0.4). Despite the higher level of serum C-peptide UCPCR0 was not different between the two groups(1.0 v 0.8, p=0.8) and UCPCR120 was lower in the CKD group (3.8 v 2.7, p=0.02). This is consistent with reduced renal clearance of C-peptide.

Figure 3

For peer review only - http://bmjopen.bmj.com/site/about/guidelines.xhtml

For peer review only

Figure 3: Scatter plots showing no association in patients with CKD between (A) fasting C-peptide and second void UCPCR (r_s 0.17, p=0.4), and (B) fasting insulin and second void UCPCR (r_s -0.17, p=0.4)

In contrast to healthy controls, there was no correlation between UCPCR0 and fasting serum C-peptide (r_s 0.17, p=0.4), insulin (r_s -0.17, p=0.4) or HOMA-IR(r_s -0.16, p=0.4), and no correlation between UCPCR120 and C-peptide (r_s=-0.09, p=1) or insulin area under the curve during the OGTT (r_s=0.26, p=0.2).(Figure 3)

DISCUSSION

Our study suggests that a fasting 2nd void morning UCPCR could be used as a marker of insulin resistance in subjects without diabetes, as long as they are not known to have chronic renal disease. The fact that this test can be done at home without the assistance of healthcare or research staff offers the opportunity to perform a simple assessment of insulin resistance in large scale epidemiological studies.

UCPCR is not a replacement for established measures of insulin resistance, but is an alternative measure of fasting insulin. Numerous population based studies have used HOMA to estimate insulin resistance [9]. Similarly, there are many studies using euglycaemic clamps, and alternative methods such as minimal model analysis to study individual patients or small groups of patients. UCPCR cannot be used as a direct substitute for these as it only measures C-peptide, and although it shows a strong correlation with HOMA2-IR, we have not validated it against the euglycaemic-hyperglycaemic clamp. The similarity of the scatter plots for UCPCR0 against HOMA2-IR and UCPCR0 against fasting insulin demonstrates the large effect that fasting insulin values have on HOMA-IR when subjects do not have abnormal fasting glucose. If fasting glucose levels are elevated, UCPCR will not correlate so well with HOMA-IR as elevated glucose will start to have an effect on the calculation. This suggests that UCPCR may only be useful as a marker of insulin resistance in populations who have normal glucose tolerance. UCPCR is a non-invasive test and does not need proximity to a laboratory for immediate sample analysis. Rather than replacing more complex measures of assessment of insulin secretion or resistance, UCPCR is an alternative

For peer review only - http://bmjopen.bmj.com/site/about/guidelines.xhtml

where serum insulin or C-peptide analysis are impractical, or the non-invasive nature of a urine test is preferred.

We collected second void fasting urine samples because we have shown this to be less variable than first void urine in people without diabetes[11]. This is because C-peptide secretion in response to the previous evening's meal will accumulate in an overnight urine sample. A second void sample adds an extra methodological step which may make sampling more difficult in large studies. It would be interesting to see how well first void urine correlated with serum insulin and C-peptide and there may be existing studies that have both serum and fasting first void urine samples available to easily test this.

A key finding of this study is that UCPCR0 and UCPCR120 were not correlated with serum C-peptide or insulin in subjects with CKD. When comparing the CKD group to the control subjects, serum C-peptide AUC was elevated in subjects with CKD whereas UCPCR120 was lower. This is explained by the reduced renal clearance of C-peptide in CKD[10], leading to higher C-peptide AUC values and lower UCPCR120 values. This impaired clearance may then explain the lack of correlation in subjects with CKD. The numbers of patients in this study were too small to compare patients with different levels of GFR, underlying causes of CKD and the presence of proteinuria. Further work will be needed to fully understand the clearance of C-peptide in people with CKD. These data suggest that UCPCR should not be used in people without diabetes who have CKD. In our previously published study on patients with T2D, mild CKD (in 23 subjects) did not alter the association between UCPCR and serum C-peptide[14]. It is possible that the presence of diabetes, more severe CKD (median eGFR 33(27-46) v 51(44-58) in Bowman's study), or relatively small numbers in both studies may explain the difference between these two sets of results. Our results suggest that further work may be needed to assess the utility of UCPCR in subjects with diabetes and renal impairment.

This study is important because of the simplicity and practicality of a UCPCR test rather than an ability to more accurately describe insulin physiology in individual subjects. Current measures of insulin secretion and sensitivity rely on serum assays of C-peptide and insulin which require access to rapid centrifugation and freezing. This limits studies to centres with these facilities and staff to use them. UCPCR could be particularly

For peer review only - http://bmjopen.bmj.com/site/about/guidelines.xhtml

important in the developing world where the diagnosis of diabetes is rising fastest and reduced facility and staffing costs associated with a posted urine sample may make large studies easier to do.

In conclusion UCPCR0 and UCPCR120 correlate with serum levels of insulin and C-peptide, and also with HOMA2 calculated insulin resistance in patients without diabetes. The practical aspects of performing UCPCR testing make it a potentially useful method for the assessment of insulin production and resistance in large epidemiology studies. Patients with CKD should be excluded from these studies.

ACKNOWLEDGEMENTS

Material costs for the project were provided by a Royal Devon and Exeter NHS Foundation Trust Small Projects Grant. ATH, BAK and BMS are core members of the NIHR Exeter Clinical Research Facility and ATH is an NIHR Senior Research Fellow. This study was supported by PenCLAHRC. RAO is funded by Diabetes UK Clinical Training Fellowship.

COMPETING INTERESTS

None

FUNDING

Royal Devon and Exeter Small Projects Grant

CONTRIBUTORSHIP

R Oram and A Rawlingson are joint first authors who wrote the manuscript, designed the study and performed the study. B Shields was the primary statistician involved in data analysis. C Bingham and R Besser both contributed to writing the manuscript, and C Bingham recruited patients from her clinics. B Knight helped design and get ethical approval for the study ,she also recruited and tested patients for the study and was involved in writing the manuscript. T McDonald performed all biochemical analysis of laboratory samples and contributed to writing the manuscript. A Hattersley is the senior author and has seen multiple drafts of the paper.

DATA SHARING

Additional data is available from Richard Oram (r.oram@exeter.ac.uk)

For peer review only

For peer review only - http://bmjopen.bmj.com/site/about/guidelines.xhtml

REFERENCES

1 Martin BC, Warram JH, Krolewski AS, et al. Role of glucose and insulin resistance in development of type 2 diabetes mellitus: results of a 25-year follow-up study. *Lancet* 1992;340(8825):925-9.
2 Bonora E, Kiechl S, Willeit J, et al. Insulin resistance as estimated by homeostasis model assessment predicts incident symptomatic cardiovascular disease in caucasian subjects from the general population: the Bruneck study. *Diabetes care* 2007;30(2):318-24.
3 Polonsky KS, Given BD, Hirsch L, et al. Quantitative study of insulin secretion and clearance in normal and obese subjects. *The Journal of clinical investigation* 1988;81(2):435-41.
4 DeFronzo RA, Tobin JD, Andres R. Glucose clamp technique: a method for quantifying insulin secretion and resistance. *Am. J. Physiol.* 1979;237:E214-23.
5 Ferrari P, Alleman Y, Shaw S, et al. Reproducibility of insulin sensitivity measured by the minimal model method. *Diabetologia* 1991;34(7):527-30.
6 Haffner SM, Miettinen H, Stern MP. The homeostasis model in the San Antonio Heart Study. *Diabetes care* 1997;20(7):1087-92.
7 Stumvoll M, Mitrakou A, Pimenta W, et al. Use of the oral glucose tolerance test to assess insulin release and insulin sensitivity. *Diabetes care* 2000;23(3):295-301.
8 Matthews DR, Hosker JP, Rudenski AS, et al. Homeostasis model assessment: insulin resistance and beta-cell function from fasting plasma glucose and insulin concentrations in man. *Diabetologia* 1985;28(7):412-9.
9 Wallace TM, Levy JC, Matthews DR. Use and abuse of HOMA modeling. *Diabetes care* 2004;27(6):1487-95.
10 Horwitz DL, Rubenstein AH, Katz AI. Quantitation of human pancreatic beta-cell function by immunoassay of C-peptide in urine. *Diabetes* 1977;26(1):30-5.
11 McDonald TJ, Knight BA, Shields BM, et al. Stability and reproducibility of a single-sample urinary C-peptide/creatinine ratio and its correlation with 24-h urinary C-peptide. *Clinical chemistry* 2009;55(11):2035-9.
12 Besser RE, Ludvigsson J, Jones AG, et al. Urine C-peptide creatinine ratio is a noninvasive alternative to the mixed-meal tolerance test in children and adults with type 1 diabetes. *Diabetes care* 2011;34(3):607-9.
13 Jones AG, Besser RE, McDonald TJ, et al. Urine C-peptide creatinine ratio is an alternative to stimulated serum C-peptide measurement in late-onset, insulin-treated diabetes. *Diabetic medicine : a journal of the British Diabetic Association* 2011;28(9):1034-8.
14 Bowman P, McDonald TJ, Shields BM, et al. Validation of a single-sample urinary C-peptide creatinine ratio as a reproducible alternative to serum C-peptide in patients with Type 2 diabetes. *Diabetic medicine : a journal of the British Diabetic Association* 2011.
15 World Health Organisation. Definition and Diagnosis of Diabetes mellitus and intermediate hyperglycaemia. 2006.
16 World Health Organisation. Use of Glycated Haemoglobin (HbA1c) in the diagnosis of Diabetes Mellitus, Abbreviated report of a WHO consultation. 2011.
17 Levey AS, Bosch JP, Lewis JB, et al. A more accurate method to estimate glomerular filtration rate from serum creatinine: a new prediction equation. Modification of Diet in Renal Disease Study Group. *Annals of internal medicine* 1999;130(6):461-70.

For peer review only

For peer review only - http://bmjopen.bmj.com/site/about/guidelines.xhtml

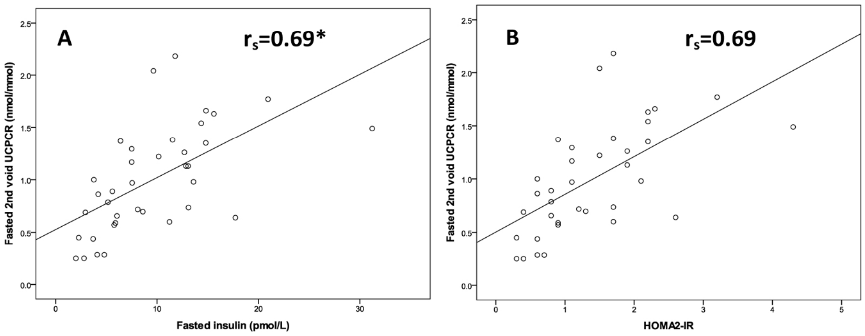

157x66mm (300 x 300 DPI)

For peer review only

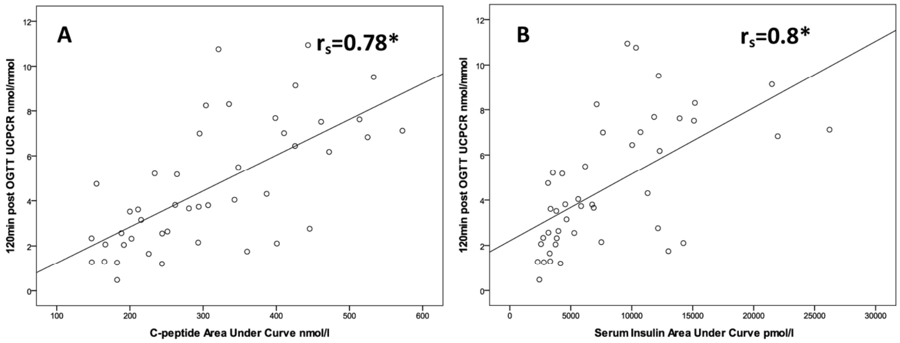

157x58mm (300 x 300 DPI)

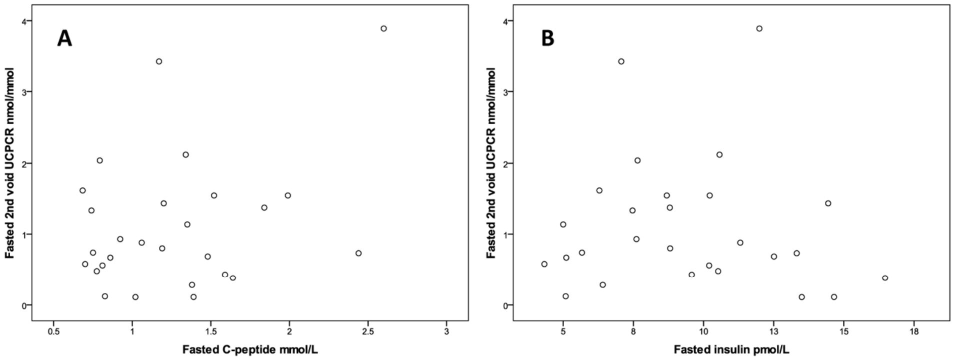

154x60mm (300 x 300 DPI)

For peer review only

STROBE Statement—checklist of items that should be included in reports of observational studies

	Item No	Recommendation
**Title and abstract**	1	Comparison of one technique to another - explained in abstract
**Introduction**		
Background/rationale	2	done
Objectives	3	done
**Methods**		
Study design	4	done
Setting	5	done
Participants	6	done
Variables	7	done
Data sources/ measurement	8*	done
Bias	9	done
Study size	10	As were looking for a correlation rather than a difference between two
Quantitative variables	11	Data were analysed continuously
Statistical methods	12	done

Continued on next page

For peer review only

**Results**		
Participants	13*	done
Descriptive data	14*	done
Outcome data	15*	*Comparison of methods using correlation. Bland Altman not possible as measuring different substance .*
Main results	16	Continuous variables analysed using nonparametric testing due to non normal distribution of data.
Other analyses	17	Analysis of CKD group shown separately
**Discussion**		
Key results	18	done
Limitations	19	done
Interpretation	20	done
Generalisability	21	discussed
**Other information**		
Funding	22	Listed in aknowledgements

*Give information separately for cases and controls in case-control studies and, if applicable, for exposed and unexposed groups in cohort and cross-sectional studies.

Note: An Explanation and Elaboration article discusses each checklist item and gives methodological background and published examples of transparent reporting. The STROBE checklist is best used in conjunction with this article (freely available on the Web sites of PLoS Medicine at http://www.plosmedicine.org/, Annals of Internal Medicine at http://www.annals.org/, and Epidemiology at http://www.epidem.com/). Information on the STROBE Initiative is available at www.strobe-statement.org.

For peer review only

For peer review only - http://bmjopen.bmj.com/site/about/guidelines.xhtml

Urine C-Peptide Creatinine Ratio can be used to assess insulin resistance and insulin production in people without diabetes.

Journal:	*BMJ Open*
Manuscript ID:	bmjopen-2013-003193.R1
Article Type:	Research
Date Submitted by the Author:	18-Jul-2013
Complete List of Authors:	Oram, Richard; University of Exeter, NIHR Exeter Clinical Research facility; Royal Devon and Exeter NHS Foundation Trust,<br>Rawlingson, Andrew; Royal Devon and Exeter NHS Foundation Trust, ; University of Exeter, NIHR Exeter Clinical Research Facility<br>Shields, Beverley; University of Exeter, NIHR Exeter Clinical Research Facility<br>Bingham, Coralie; Royal Devon and Exeter NHS Foundation Trust,<br>Besser, Rachel; University of Exeter, NIHR Exeter Clinical Research Facility<br>McDonald, Timothy; Royal Devon and Exeter NHS Foundation Trust, ; University of Exeter, NIHR Exeter Clinical Research Facility<br>Knight, Bridget; University of Exeter, NIHR Exeter Clinical Research Facility<br>Hattersley, Andrew; University of Exeter, NIHR Exeter Clinical Research Facility
**Primary Subject Heading**:	Diabetes and endocrinology
Secondary Subject Heading:	Epidemiology
Keywords:	Epidemiology < TROPICAL MEDICINE, DIABETES & ENDOCRINOLOGY, STATISTICS & RESEARCH METHODS

SCHOLARONE™
Manuscripts

Urine C-Peptide Creatinine Ratio can be used to assess insulin resistance and insulin production in people without diabetes.

*Richard A Oram[1,2], *Andrew Rawlingson[1,2], Beverley M Shields[1], Coralie. Bingham[2], Rachel EJ Besser[1],Tim J McDonald[1,2], Bridget A Knight[1], Andrew T Hattersley[1]

*Joint first authors

1. NIHR Exeter Clinical Research Facility, University of Exeter Medical School, Royal Devon & Exeter NHS Foundation Trust, Barrack Road, Exeter, UK.
2. Royal Devon & Exeter NHS Foundation Trust, Barrack Road, Exeter, UK

Word Count: Abstract 200w, article 2241w

Key words: UCPCR, HOMA, Insulin Resistance, C-peptide/urine, Insulin/secretion

Corresponding Author:

Dr Richard Oram

r.oram@exeter.ac.uk

Tel: +44 1392 406807
Fax: +44 1392 406767

Level 2
NIHR Exeter Clinical Research Facility
University of Exeter Medical School
Barrack Road
Exeter
UK
EX2 5DW

For peer review only

For peer review only - http://bmjopen.bmj.com/site/about/guidelines.xhtml

ARTICLE FOCUS

- Large epidemiology studies often use a serum measurement of fasting insulin and glucose to measure insulin resistance.
- Measurement of Urine C-peptide creatinine ratio (UCPCR) is a non-invasive measure of insulin production that can be posted from home.

KEY MESSAGES

- Urine C-peptide creatinine ratio strongly correlates with serum insulin levels, and HOMA calculated insulin resistance in people without diabetes.
- Epidemiology studies of insulin resistance can now be performed without needing blood testing, using a posted urine sample.

STRENGTHS AND LIMITATIONS

- This study uses both a clinical research facility setting, and samples sent from home to demonstrate that UCPCR can be used in healthy volunteers.
- UCPCR is compared to other epidemiological measures of insulin resistance such as fasting insulin and HOMA.
- UCPCR is not valid in people with Chronic Kidney Disease stages 3-5

For peer review only

ABSTRACT

OBJECTIVES
Current assessment of insulin resistance(IR) in epidemiology studies relies on blood measurement of C-peptide or insulin. Urine C-peptide creatinine ratio (UCPCR) can be posted from home unaided. It is validated against serum measures of insulin in people with diabetes. We tested whether UCPCR could be a surrogate measure of IR by examining the correlation of UCPCR with serum insulin, C-peptide and HOMA2-IR in subjects without diabetes, and in subjects with Chronic Kidney Disease (CKD).

DESIGN
Observational study

SETTING
Single centre Clinical Research Facility

PARTICIPANTS
37 healthy volunteers and 30 patients with CKD (GFR 15-60) were recruited.

PRIMARY AND SECONDARY ENDPOINTS
Serum insulin, C-peptide and glucose at fasting(0), 30, 60, 90 and 120 minutes were measured during an oral glucose tolerance test (OGTT). Second void fasting UCPCR and 120 minutes post OGTT UCPCR were collected. HOMA2-IR was calculated using fasting insulin and glucose. Associations between UCPCR and serum measures were assessed using Spearman's correlations.

RESULTS
In healthy volunteers, fasting second void UCPCR strongly correlated with serum insulin (r_s=0.69, p<0.0001), C-peptide(r_s=0.73, p<0.0001) and HOMA2-IR (r_s=-0.69, p<0.0001). 120min post OGTT UCPCR correlated strongly with C-peptide and insulin area under the curve. In patients with CKD, UCPCR did not correlate with serum C-peptide, insulin or HOMA2-IR.

CONCLUSION
In subjects with normal renal function, UCPCR may be a simple, practical method for the assessment of IR in epidemiology studies.

For peer review only

BACKGROUND

Insulin resistance has been shown to be a significant predictor for the development of diabetes and for cardiovascular risk[1,2]. Understanding the epidemiology of insulin resistance is important in the identification of patients at risk of type 2 diabetes (T2D) and vascular disease, and for the study of prevention. The optimum individual method to assess insulin physiology uses glucose disposal rate during hyperinsulinaemic-euglycaemic clamp studies[3,4], which require infusions of both insulin and glucose and cannot be used at a population level. Minimal model analysis of glucose and insulin levels during intravenous or oral glucose loading allows assessment without the use of intravenous insulin, but still necessitates multiple blood samples[5]. Fasting assessments of insulin alone, or with measures of glucose have been used as a more simple method to study insulin resistance[6], and have been validated against other more invasive tests[7]. One widely used approach that allows for variation in the fasting glucose is the Homeostasis Model Assessment (HOMA, http://www.dtu.ox.ac.uk/homacalculator/index.php)[8,9] that models fasting serum glucose, and insulin or C-peptide levels to calculate a measure of insulin resistance. HOMA requires that a fasting blood sample is taken and the sample is relatively rapidly processed within 24 hours[10]. This means an appointment with healthcare or research staff is still required and this is not always readily available for some large epidemiological studies.

An alternative method to blood sampling, which allows samples to be provided without outside assistance, is to measure urinary C-peptide. C-peptide is secreted in equimolar amounts to insulin but unlike insulin, is filtered by the kidney with 5% excreted unchanged in the urine, making urinary measures possible[11]. We have recently demonstrated that C-peptide is measureable, reproducible and stable in urine for up to 72 hours in boric acid preservative (allowing postage from primary care or from home)[12]. Measuring C-peptide as a ratio against creatinine allows the use of a single spot urine sample by accounting for dilution in the same way as protein creatinine ratio. In patients with type 1 diabetes (T1D) and T2D, 2 hour urine C-peptide creatinine ratio (UCPCR) is highly correlated to 90 minute serum C-peptide in the standard Mixed Meal Tolerance Test[13,14]. We have also shown that in patients with T2D and mild chronic kidney disease (CKD), the correlation between serum C-peptide and urine is maintained[15]. As fasting serum insulin or C-peptide alone is a helpful marker of insulin resistance in people without diabetes, it may be that UCPCR could also be used in this manner.

For peer review only - http://bmjopen.bmj.com/site/about/guidelines.xhtml

If UCPCR can be used in people without diabetes this practical method could allow large scale, population based assessment of insulin resistance without needing a blood sample to be taken. We aimed to test whether UCPCR could be used as a surrogate measure of insulin resistance in epidemiological studies by examining the correlation of UCPCR with fasting serum insulin, C-peptide and HOMA2-IR in subjects without diabetes. As a secondary outcome we tested whether stimulated UCPCR could be used as a marker of insulin secretion during an oral glucose tolerance test. We also wanted to see if the correlations were maintained in subjects with chronic kidney disease.

METHODS

Study participants

2 groups were recruited from December 2009 to May 2010:

37 healthy controls (22 female) with normal renal function (eGFR>60ml $min^{-1}m^{-2}$), and normal glucose tolerance[16,17] were recruited from research volunteer databases in Devon.

30 patients (8 female) with normal glucose tolerance and a clinical diagnosis of CKD stage 3 or greater (MDRD eGFR<60ml $min^{-1}m^{-2}$) (www.renal.org/CKDguide/full/UKCKDfull.pdf) were recruited from general nephrology clinics at the Royal Devon and Exeter Hospital. Patients on renal replacement therapy (either dialysis or transplant) were excluded from the study.

Table 1		
	Normal renal function group	CKD group
Total subjects	37	30
Female	22	8
Age (years)	50 (29-67)	65(52-71)
BMI (Kg/m^2)	27.0(23.5-33.0)	26.4(24.1-28.6)
HbA1c (%)	5.7(5.4-6.0)	5.9(5.6-6.1)
Fasting Blood Glucose (mmol/L)	4.8(4.5-5.1)	5.0(4.5-5.3)
Creatinine (mmol/L)	77(66-84)	195(134-231)
MDRD eGFR (ml/min/1.73m^2)	88(76-101)	32(26-46)

Table 1 – Cohort Characteristics. Data are presented as median (interquartile range)

All studies were performed with approval from the South West 2 Research Ethics Committee.

For peer review only - http://bmjopen.bmj.com/site/about/guidelines.xhtml

Clinical sampling

Participants fasted from midnight prior to their visit and emptied their bladder on waking (this first-void urine was not collected). Demographic data, past medical and drug history were recorded. Baseline fasting blood samples were collected for routine analysis of glucose, HbA1c and renal function. A second urine sample (second-void fasting) was collected immediately prior to OGTT for measurement of UCPCR(UCPCR0)[6].

In a standard OGTT (75 g glucose), blood samples were collected at 30, 60 90 and 120 minutes. A further urine sample was collected for UCPCR analysis at 120 minutes (UCPCR120). Blood samples were immediately centrifuged and separated. Serum and urine samples were initially stored at -20℃ then transferred and stored at -80℃ within 1 week. Serum samples were subsequently analysed for insulin, C-peptide, and glucose. Urine samples were analysed for C-peptide and creatinine and a urine C-peptide creatinine ratio was calculated.

Biochemical analysis

Urine and serum C-peptide analysis were performed by electro-chemiluminescence immunoassay (Roche Diagnostics E170 C-peptide assay). All urine samples were pre-diluted 1:10 with equine serum albumin (diluent multianalyte, Roche Diagnostics, Mannheim, Germany). Serum insulin analysis was performed by electro-chemiluminescence immunoassay (Roche Diagnostics E170 C-peptide assay). Glucose and creatinine were analysed on the Roche P800 modular platforms. All analysis was performed in the Department of Chemical Pathology, Royal Devon and Exeter Hospital. eGFR was calculated using 4-variable MDRD formula[18].

Data analysis

Serial serum C-peptide, insulin and glucose measurements were used to calculate area under the curve (AUC) for each parameter. Insulin resistance (HOMA2-IR) was derived from fasting glucose and insulin [http://www.dtu.ox.ac.uk/homacalculator/index.php]. Associations between second void UCPCR0 and stimulated UCPCR 120 with serum C-peptide, insulin, HOMA2-IR were assessed using Spearman correlations. Analyses were performed separately for the group with CKD and the group without CKD. Data for

For peer review only - http://bmjopen.bmj.com/site/about/guidelines.xhtml

UCPCR were non-normally distributed so non-parametric statistical testing was used for analysis

RESULTS

A summary of the characteristics of the study group is shown in Table 1. 3 subjects with CKD had serum C-peptide samples that were not analysed due to sampling problems, their results have been included in analyses excluding those involving C-peptide values.

Table 2		
	Normal renal function group (n=38)	CKD group (n=30)
Fasting C-Peptide (nmol/L)	0.7(0.5-1.0)	1.2(0.8-1.6)
Fasting Insulin (pmol/L)	8.1(5.0-13.1)	8.8(6.4-12.0)
C-Peptide Area Under Curve(nmol/L)	294(207-405)	457(371-550)
Insulin Area Under Curve (pmol/L)	6180(3641-11994)	7685(5050-9597)
UCPCR0 (nmol/mmol)	1.0(0.6-1.4)	0.914(0.5-1.5)
UCPCR120 (nmol/mmol)	3.8(2.3-7.0)	2.8(0.9-4.0)
HOMA2-IR	1.2 (0.8-1.9)	1.3 (0.9-1.7)

Table 2 – Median (Interquartile range) serum insulin, C-peptide, UCPCR and HOMA-IR

Fasting 2nd void UCPCR strongly correlated with serum insulin, C-peptide and HOMA2-IR in people without chronic kidney disease.

In subjects without renal disease fasting second void UCPCR0 strongly correlated with serum insulin (r_s=0.69, p<0.0001), C-peptide(r_s=0.73, p<0.0001) and HOMA2-IR (r_s=-0.69, p<0.0001). Age and BMI also correlated with HOMA2-IR (r=0.50 and 0.52 respectively, p<0.0001 for both). Scatter plots with Spearman's correlations and regression lines are shown in Figure1.

For peer review only

FIGURE LEGENDS

Figure 1 Scatter plots showing fasting second void UCPCR (UCPCR0) was strongly correlated to fasting serum insulin (A) and HOMA2-IR (B) in 37 people with normal renal function. Regression line Spearman's r_s correlations shown. *=p<0.0001

Stimulated UCPCR values were correlated with stimulated values of serum insulin and C-peptide, in people without chronic kidney disease.

After an OGTT, UCPCR120 values were higher than UCPCR0 (3.8 v 1.0 nmol/mmol, p<0.0001)(Table 2). UCPCR120 correlated with serum insulin (r_s0.78, p<0.0001) and C-peptide area under the curve (r_s0.8, p<0.0001). Scatter plots with Spearman's correlations and regression lines are shown in Figure 2.

Figure 2 Scatter plots showing 120 minute post OGTT UCPCR (UCPCR120) was strongly correlated to serum C-peptide (A) and insulin (B) area under the curve in 37 people with normal renal function. Regression line Spearman's r_s correlations shown. *=p<0.0001

In patients with CKD, UCPCR does not correlate with serum C-peptide, insulin or HOMA2-IR

In patients with CKD, median fasting (1.2 v 0.7 nmol/L p<0.0001) and stimulated (457 v 294 nmol/L, p<0.0001)serum C-peptide measures were higher than the subjects without CKD, but serum insulin levels were not different (7685 v 6180 , p=0.4). Despite the higher level of serum C-peptide UCPCR0 was not different between the two groups(1.0 v 0.8, p=0.8) and UCPCR120 was lower in the CKD group (3.8 v 2.7, p=0.02). This is consistent with reduced renal clearance of C-peptide.

Figure 3: Scatter plots showing no association in patients with CKD between (A) fasting C-peptide and second void UCPCR (r_s 0.17, p=0.4), and (B) fasting insulin and second void UCPCR (r_s -0.17, p=0.4)

In contrast to healthy controls, there was no correlation between UCPCR0 and fasting serum C-peptide (r_s 0.17, p=0.4), insulin (r_s -0.17, p=0.4) or HOMA-IR(r_s -0.16, p=0.4), and no correlation between UCPCR120 and C-peptide (r_s=-0.09, p=1) or insulin area under the curve during the OGTT (r_s=0.26, p=0.2).(Figure 3)

For peer review only - http://bmjopen.bmj.com/site/about/guidelines.xhtml

DISCUSSION

Our study suggests that a fasting 2nd void morning UCPCR could be used as a marker of insulin resistance in subjects without diabetes, as long as they are known not to have chronic renal disease. The fact that this test can be done at home without the assistance of healthcare or research staff offers the opportunity to perform a simple assessment of insulin resistance in large scale epidemiological studies.

UCPCR is not a replacement for established measures of insulin resistance, but is an alternative measure of fasting insulin. Numerous population based studies have used HOMA to estimate insulin resistance [9]. Similarly, there are many studies using euglycaemic clamps, and alternative methods such as minimal model analysis to study individual patients or small groups of patients. UCPCR cannot be used as a direct substitute for these as it only measures C-peptide, and although it shows a strong correlation with HOMA2-IR, we have not validated it against the euglycaemic-hyperglycaemic clamp. The similarity of the scatter plots for UCPCR0 against HOMA2-IR and UCPCR0 against fasting insulin demonstrates the large effect that fasting insulin values have on HOMA-IR when subjects do not have abnormal fasting glucose. If fasting glucose levels are elevated, UCPCR will not correlate so well with HOMA-IR as elevated glucose will start to have an effect on the calculation. This suggests that UCPCR may only be useful as a marker of insulin resistance in populations who have normal glucose tolerance. There was a correlation between HOMA2-IR, age and BMI in our study, but the variance explained by these simple measure was less than UCPCR0 (r^2=0.27 for BMI v r^2=0.48 for UCPCR0), suggesting it has additional benefit over these measures. UCPCR is a non-invasive test and does not need proximity to a laboratory for immediate sample analysis. Rather than replacing more complex measures of assessment of insulin secretion or resistance, UCPCR is an alternative where serum insulin or C-peptide analysis are impractical, or the non-invasive nature of a urine test is preferred.

We collected second void fasting urine samples because we have shown this to be less variable than first void urine in people without diabetes[12]. This is because C-peptide secretion in response to the previous evening's meal will accumulate in an overnight urine sample. A second void sample adds an extra methodological step which may make sampling more difficult in large studies. It would be interesting to see how well

For peer review only - http://bmjopen.bmj.com/site/about/guidelines.xhtml

first void urine correlated with serum insulin and C-peptide and there may be existing studies that have both serum and fasting first void urine samples available to easily test this.

A key finding of this study is that UCPCR0 and UCPCR120 were not correlated with serum C-peptide or insulin in subjects with CKD. When comparing the CKD group to the control subjects, serum C-peptide AUC was elevated in subjects with CKD whereas UCPCR120 was lower. This is explained by the reduced renal clearance of C-peptide in CKD[11], leading to higher C-peptide AUC values and lower UCPCR120 values. This impaired clearance may then explain the lack of correlation in subjects with CKD. The numbers of patients in this study were too small to compare patients with different levels of GFR, underlying causes of CKD and the presence of proteinuria. Further work will be needed to fully understand the clearance of C-peptide in people with CKD. These data suggest that UCPCR should not be used in people without diabetes who have CKD. In our previously published study on patients with T2D, mild CKD (in 23 subjects) did not alter the association between UCPCR and serum C-peptide[15]. It is possible that the presence of diabetes, more severe CKD (median eGFR 33(27-46) v 51(44-58) in Bowman's study), or relatively small numbers in both studies may explain the difference between these two sets of results. Our results suggest that further work may be needed to assess the utility of UCPCR in subjects with diabetes and renal impairment.

This study is important because of the simplicity and practicality of a UCPCR test rather than an ability to more accurately describe insulin physiology in individual subjects. Current measures of insulin secretion and sensitivity rely on serum assays of C-peptide and insulin which require access to centrifugation and freezing within 24 hours. This limits studies to centres with these facilities and staff to use them. UCPCR could be particularly useful in the developing world where the diagnosis of diabetes is rising fastest and reduced facility and staffing costs associated with a posted urine sample may make large studies easier to do. Given the results with CKD and the effect of elevated glucose levels on HOMA calculated insulin resistance, UCPCR may be most useful in young or middle aged populations where the background prevalence of CKD and diabetes is low.

In conclusion UCPCR0 and UCPCR120 correlate with serum levels of insulin and C-peptide, and also with HOMA2 calculated insulin resistance in patients without

For peer review only - http://bmjopen.bmj.com/site/about/guidelines.xhtml

diabetes. The practical aspects of performing UCPCR testing make it a potentially useful method for the assessment of insulin production and resistance in large epidemiology studies. Patients with CKD should be excluded from these studies.

ACKNOWLEDGEMENTS

Material costs for the project were provided by a Royal Devon and Exeter NHS Foundation Trust Small Projects Grant. ATH, BAK and BMS are core members of the NIHR Exeter Clinical Research Facility and ATH is an NIHR Senior Research Fellow. This study was supported by PenCLAHRC. RAO is funded by Diabetes UK Clinical Training Fellowship.

Competing Interests

None

Contributorship

R Oram and A Rawlingson are joint first authors who wrote the manuscript, designed the study and performed the study. B Shields was the primary statistician involved in data analysis. C Bingham and R Besser both contributed to writing the manuscript, and C Bingham recruited patients from her clinics. B Knight helped design and get ethical approval for the study ,she also recruited and tested patients for the study and was involved in writing the manuscript. T McDonald performed all biochemical analysis of laboratory samples and contributed to writing the manuscript. A Hattersley is the senior author and has seen multiple drafts of the paper.

Funding

Royal Devon and Exeter Small Projects Grant

Data sharing

Additional data is available from Richard Oram (including individual anonymous results on UCPCR, serum blood measurements, HOMA2IR calculations and baseline characteristics of cohort. For extra information please write to:-r.oram@exeter.ac.uk

For peer review only - http://bmjopen.bmj.com/site/about/guidelines.xhtml

For peer review only

REFERENCES

1. Martin BC, Warram JH, Krolewski AS, et al. Role of glucose and insulin resistance in development of type 2 diabetes mellitus: results of a 25-year follow-up study. *Lancet* 1992;340(8825):925-9.
2. Bonora E, Kiechl S, Willeit J, et al. Insulin resistance as estimated by homeostasis model assessment predicts incident symptomatic cardiovascular disease in caucasian subjects from the general population: the Bruneck study. *Diabetes care* 2007;30(2):318-24.
3. Polonsky KS, Given BD, Hirsch L, et al. Quantitative study of insulin secretion and clearance in normal and obese subjects. *The Journal of clinical investigation* 1988;81(2):435-41.
4. DeFronzo RA, Tobin JD, Andres R. Glucose clamp technique: a method for quantifying insulin secretion and resistance. *Am. J. Physiol.* 1979;237:E214-23.
5. Ferrari P, Alleman Y, Shaw S, et al. Reproducibility of insulin sensitivity measured by the minimal model method. *Diabetologia* 1991;34(7):527-30.
6. Haffner SM, Miettinen H, Stern MP. The homeostasis model in the San Antonio Heart Study. *Diabetes care* 1997;20(7):1087-92.
7. Stumvoll M, Mitrakou A, Pimenta W, et al. Use of the oral glucose tolerance test to assess insulin release and insulin sensitivity. *Diabetes care* 2000;23(3):295-301.
8. Matthews DR, Hosker JP, Rudenski AS, et al. Homeostasis model assessment: insulin resistance and beta-cell function from fasting plasma glucose and insulin concentrations in man. *Diabetologia* 1985;28(7):412-9.
9. Wallace TM, Levy JC, Matthews DR. Use and abuse of HOMA modeling. *Diabetes care* 2004;27(6):1487-95.
10. McDonald TJ, Perry MH, Peake RW, et al. EDTA improves stability of whole blood C-peptide and insulin to over 24 hours at room temperature. *PLoS One* 2012;7(7):e42084.
11. Horwitz DL, Rubenstein AH, Katz AI. Quantitation of human pancreatic beta-cell function by immunoassay of C-peptide in urine. *Diabetes* 1977;26(1):30-5.
12. McDonald TJ, Knight BA, Shields BM, et al. Stability and reproducibility of a single-sample urinary C-peptide/creatinine ratio and its correlation with 24-h urinary C-peptide. *Clinical chemistry* 2009;55(11):2035-9.
13. Besser RE, Ludvigsson J, Jones AG, et al. Urine C-peptide creatinine ratio is a noninvasive alternative to the mixed-meal tolerance test in children and adults with type 1 diabetes. *Diabetes care* 2011;34(3):607-9.
14. Jones AG, Besser RE, McDonald TJ, et al. Urine C-peptide creatinine ratio is an alternative to stimulated serum C-peptide measurement in late-onset, insulin-treated diabetes. *Diabetic medicine : a journal of the British Diabetic Association* 2011;28(9):1034-8.
15. Bowman P, McDonald TJ, Shields BM, et al. Validation of a single-sample urinary C-peptide creatinine ratio as a reproducible alternative to serum C-peptide in patients with Type 2 diabetes. *Diabet Med* 2012;29(1):90-3.
16. Organisation WH. Definition and Diagnosis of Diabetes mellitus and intermediate hyperglycaemia. 2006.
17. Organisation WH. Use of Glycated Haemoglobin (HbA1c) in the diagnosis of Diabetes Mellitus, Abbreviated report of a WHO consultation. 2011.

For peer review only

18. Levey AS, Bosch JP, Lewis JB, et al. A more accurate method to estimate glomerular filtration rate from serum creatinine: a new prediction equation. Modification of Diet in Renal Disease Study Group. *Annals of internal medicine* 1999;130(6):461-70.

For peer review only

For peer review only - http://bmjopen.bmj.com/site/about/guidelines.xhtml

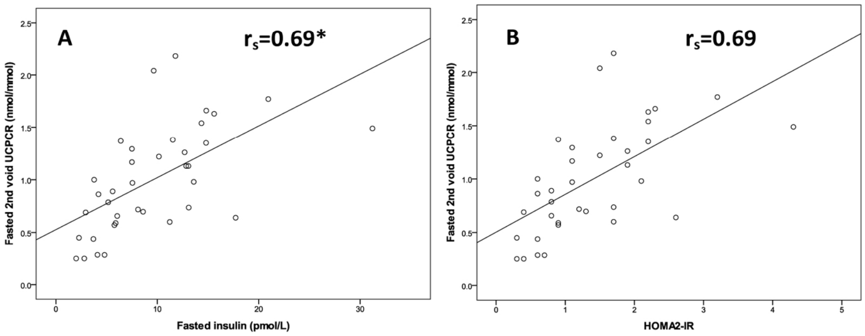

157x66mm (300 x 300 DPI)

For peer review only

Urine C-Peptide Creatinine Ratio can be used t insulin resistance and insulin production in people without diabetes.

***Richard A Oram[1,2], *Andrew Rawlingson[1,2], Beverley M Shields[1], Coralie. Bingham[2], Rachel EJ Besser[1],Tim J McDonald[1,2], Bridget A Knight[1], Andrew T Hattersley[1]**

*Joint first authors

1. NIHR Exeter Clinical Research Facility, University of Exeter Medical School, Royal Devon & Exeter NHS Foundation Trust, Barrack Road, Exeter, UK.
2. Royal Devon & Exeter NHS Foundation Trust, Barrack Road, Exeter, UK

For peer review only

Word Count: Abstract 200w, article 2241w

Key words: UCPCR, HOMA, Insulin Resistance, C-peptide/urine, Insulin/secretion

Corresponding Author:

Dr Richard Oram

r.oram@exeter.ac.uk

Tel: +44 1392 406807
Fax: +44 1392 406767

Level 2
NIHR Exeter Clinical Research Facility
University of Exeter Medical School
Barrack Road
Exeter
UK
EX2 5DW

For peer review only - http://bmjopen.bmj.com/site/about/guidelines.xhtml

ARTICLE FOCUS

- Large epidemiology studies often use a serum measurement of fasting insulin and glucose to measure insulin resistance.
- Measurement of Urine C-peptide creatinine ratio (UCPCR) is a non-invasive measure of insulin production that can be posted from home.

KEY MESSAGES

- Urine C-peptide creatinine ratio strongly correlates with serum insulin levels, and HOMA calculated insulin resistance in people without diabetes.
- Epidemiology studies of insulin resistance can now be performed without needing blood testing, using a posted urine sample.

STRENGTHS AND LIMITATIONS

- This study uses both a clinical research facility setting, and samples sent from home to demonstrate that UCPCR can be used in healthy volunteers.
- UCPCR is compared to other epidemiological measures of insulin resistance such as fasting insulin and HOMA.
- UCPCR is not valid in people with Chronic Kidney Disease stages 3-5

For peer review only

ABSTRACT

OBJECTIVES
Current assessment of insulin resistance(IR) in epidemiology studies relies on blood measurement of C-peptide or insulin. Urine C-peptide creatinine ratio (UCPCR) can be posted from home unaided. It is validated against serum measures of insulin in people with diabetes. We tested whether UCPCR could be a surrogate measure of IR by examining the correlation of UCPCR with serum insulin, C-peptide and HOMA2-IR in subjects without diabetes, and in subjects with Chronic Kidney Disease (CKD).

DESIGN
Observational study

SETTING
Single centre Clinical Research Facility

PARTICIPANTS
37 healthy volunteers and 30 patients with CKD (GFR 15-60) were recruited.

PRIMARY AND SECONDARY ENDPOINTS
Serum insulin, C-peptide and glucose at fasting(0), 30, 60, 90 and 120 minutes were measured during an oral glucose tolerance test (OGTT). Second void fasting UCPCR and 120 minutes post OGTT UCPCR were collected. HOMA2-IR was calculated using fasting insulin and glucose. Associations between UCPCR and serum measures were assessed using Spearman's correlations.

RESULTS
In healthy volunteers, fasting second void UCPCR strongly correlated with serum insulin (r_s=0.69, p<0.0001), C-peptide(r_s=0.73, p<0.0001) and HOMA2-IR (r_s=-0.69, p<0.0001). 120min post OGTT UCPCR correlated strongly with C-peptide and insulin area under the curve. In patients with CKD, UCPCR did not correlate with serum C-peptide, insulin or HOMA2-IR.

CONCLUSION
In subjects with normal renal function, UCPCR may be a simple, practical method for the assessment of IR in epidemiology studies.

For peer review only - http://bmjopen.bmj.com/site/about/guidelines.xhtml

BACKGROUND

Insulin resistance has been shown to be a significant predictor for the development of diabetes and for cardiovascular risk[1,2]. Understanding the epidemiology of insulin resistance is important in the identification of patients at risk of type 2 diabetes (T2D) and vascular disease, and for the study of prevention. The optimum individual method to assess insulin physiology uses glucose disposal rate during hyperinsulinaemic-euglycaemic clamp studies[3,4], which require infusions of both insulin and glucose and cannot be used at a population level. Minimal model analysis of glucose and insulin levels during intravenous or oral glucose loading allows assessment without the use of intravenous insulin, but still necessitates multiple blood samples[5]. Fasting assessments of insulin alone, or with measures of glucose have been used as a more simple method to study insulin resistance[6], and have been validated against other more invasive tests[7]. One widely used approach that allows for variation in the fasting glucose is the Homeostasis Model Assessment (HOMA, http://www.dtu.ox.ac.uk/homacalculator/index.php)[8,9] that models fasting serum glucose, and insulin or C-peptide levels to calculate a measure of insulin resistance. HOMA requires that a fasting blood sample is taken and the sample is relatively rapidly processed within 24 hours[10]. This means an appointment with healthcare or research staff is still required and this is not always readily available for some large epidemiological studies.

An alternative method to blood sampling, which allows samples to be provided without outside assistance, is to measure urinary C-peptide. C-peptide is secreted in equimolar amounts to insulin but unlike insulin, is filtered by the kidney with 5% excreted unchanged in the urine, making urinary measures possible[11]. We have recently demonstrated that C-peptide is measureable, reproducible and stable in urine for up to 72 hours in boric acid preservative (allowing postage from primary care or from home)[12]. Measuring C-peptide as a ratio against creatinine allows the use of a single spot urine sample by accounting for dilution in the same way as protein creatinine ratio. In patients with type 1 diabetes (T1D) and T2D, 2 hour urine C-peptide creatinine ratio (UCPCR) is highly correlated to 90 minute serum C-peptide in the standard Mixed Meal Tolerance Test[13,14]. We have also shown that in patients with T2D and mild chronic kidney disease (CKD), the correlation between serum C-peptide and urine is maintained[15]. As fasting serum insulin or C-peptide alone is a helpful marker of insulin resistance in people without diabetes, it may be that UCPCR could also be used in this manner.

For peer review only - http://bmjopen.bmj.com/site/about/guidelines.xhtml

If UCPCR can be used in people without diabetes this practical method could allow large scale, population based assessment of insulin resistance without needing a blood sample to be taken. We aimed to test whether UCPCR could be used as a surrogate measure of insulin resistance in epidemiological studies by examining the correlation of UCPCR with fasting serum insulin, C-peptide and HOMA2-IR in subjects without diabetes. As a secondary outcome we tested whether stimulated UCPCR could be used as a marker of insulin secretion during an oral glucose tolerance test. We also wanted to see if the correlations were maintained in subjects with chronic kidney disease.

METHODS

Study participants

2 groups were recruited from December 2009 to May 2010:

37 healthy controls (22 female) with normal renal function (eGFR>60ml $min^{-1}m^{-2}$), and normal glucose tolerance[16,17] were recruited from research volunteer databases in Devon.

30 patients (8 female) with normal glucose tolerance and a clinical diagnosis of CKD stage 3 or greater (MDRD eGFR<60ml $min^{-1}m^{-2}$) (www.renal.org/CKDguide/full/UKCKDfull.pdf) were recruited from general nephrology clinics at the Royal Devon and Exeter Hospital. Patients on renal replacement therapy (either dialysis or transplant) were excluded from the study.

Table 1		
	Normal renal function group	CKD group
Total subjects	37	30
Female	22	8
Age (years)	50 (29-67)	65(52-71)
BMI (Kg/m^2)	27.0(23.5-33.0)	26.4(24.1-28.6)
HbA1c (%)	5.7(5.4-6.0)	5.9(5.6-6.1)
Fasting Blood Glucose (mmol/L)	4.8(4.5-5.1)	5.0(4.5-5.3)
Creatinine (mmol/L)	77(66-84)	195(134-231)
MDRD eGFR ($ml/min/1.73m^2$)	88(76-101)	32(26-46)

Table 1 – Cohort Characteristics. Data are presented as median (interquartile range)

All studies were performed with approval from the South West 2 Research Ethics Committee.

For peer review only - http://bmjopen.bmj.com/site/about/guidelines.xhtml

Clinical sampling

Participants fasted from midnight prior to their visit and emptied their bladder on waking (this first-void urine was not collected). Demographic data, past medical and drug history were recorded. Baseline fasting blood samples were collected for routine analysis of glucose, HbA1c and renal function. A second urine sample (second-void fasting) was collected immediately prior to OGTT for measurement of UCPCR(UCPCR0)[6].

In a standard OGTT (75 g glucose), blood samples were collected at 30, 60 90 and 120 minutes. A further urine sample was collected for UCPCR analysis at 120 minutes (UCPCR120). Blood samples were immediately centrifuged and separated. Serum and urine samples were initially stored at -20°C then transferred and stored at -80°C within 1 week. Serum samples were subsequently analysed for insulin, C-peptide, and glucose. Urine samples were analysed for C-peptide and creatinine and a urine C-peptide creatinine ratio was calculated.

Biochemical analysis

Urine and serum C-peptide analysis were performed by electro-chemiluminescence immunoassay (Roche Diagnostics E170 C-peptide assay). All urine samples were pre-diluted 1:10 with equine serum albumin (diluent multianalyte, Roche Diagnostics, Mannheim, Germany). Serum insulin analysis was performed by electro-chemiluminescence immunoassay (Roche Diagnostics E170 C-peptide assay). Glucose and creatinine were analysed on the Roche P800 modular platforms. All analysis was performed in the Department of Chemical Pathology, Royal Devon and Exeter Hospital. eGFR was calculated using 4-variable MDRD formula[18].

Data analysis

Serial serum C-peptide, insulin and glucose measurements were used to calculate area under the curve (AUC) for each parameter. Insulin resistance (HOMA2-IR) was derived from fasting glucose and insulin [http://www.dtu.ox.ac.uk/homacalculator/index.php]. Associations between second void UCPCR0 and stimulated UCPCR 120 with serum C-peptide, insulin, HOMA2-IR were assessed using Spearman correlations. Analyses were performed separately for the group with CKD and the group without CKD. Data for

For peer review only - http://bmjopen.bmj.com/site/about/guidelines.xhtml

UCPCR were non-normally distributed so non-parametric statistical testing was used for analysis

RESULTS

A summary of the characteristics of the study group is shown in Table 1. 3 subjects with CKD had serum C-peptide samples that were not analysed due to sampling problems, their results have been included in analyses excluding those involving C-peptide values.

Table 2		
	Normal renal function group (n=38)	CKD group (n=30)
Fasting C-Peptide (nmol/L)	0.7(0.5-1.0)	1.2(0.8-1.6)
Fasting Insulin (pmol/L)	8.1(5.0-13.1)	8.8(6.4-12.0)
C-Peptide Area Under Curve(nmol/L)	294(207-405)	457(371-550)
Insulin Area Under Curve (pmol/L)	6180(3641-11994)	7685(5050-9597)
UCPCR0 (nmol/mmol)	1.0(0.6-1.4)	0.914(0.5-1.5)
UCPCR120 (nmol/mmol)	3.8(2.3-7.0)	2.8(0.9-4.0)
HOMA2-IR	1.2 (0.8-1.9)	1.3 (0.9-1.7)

Table 2 – Median (Interquartile range) serum insulin, C-peptide, UCPCR and HOMA-IR

Fasting 2nd void UCPCR strongly correlated with serum insulin, C-peptide and HOMA2-IR in people without chronic kidney disease.

In subjects without renal disease fasting second void UCPCR0 strongly correlated with serum insulin (r_s=0.69, p<0.0001), C-peptide(r_s=0.73, p<0.0001) and HOMA2-IR (r_s=-0.69, p<0.0001). Age and BMI also correlated with HOMA2-IR (r=0.50 and 0.52 respectively, p<0.0001 for both). Scatter plots with Spearman's correlations and regression lines are shown in Figure1.

Figure 1

For peer review only - http://bmjopen.bmj.com/site/about/guidelines.xhtml

Figure 1 Scatter plots showing fasting second void UCPCR (UCPCR0) was strongly correlated to fasting serum insulin (A) and HOMA2-IR (B) in 37 people with normal renal function. Regression line Spearman's r_s correlations shown. *=p<0.0001

Stimulated UCPCR values were correlated with stimulated values of serum insulin and C-peptide, in people without chronic kidney disease.

After an OGTT, UCPCR120 values were higher than UCPCR0 (3.8 v 1.0 nmol/mmol, p<0.0001)(Table 2). UCPCR120 correlated with serum insulin (r_s0.78, p<0.0001) and C-peptide area under the curve (r_s0.8, p<0.0001). Scatter plots with Spearman's correlations and regression lines are shown in Figure 2.

Figure 2

Figure 2 Scatter plots showing 120 minute post OGTT UCPCR (UCPCR120) was strongly correlated to serum C-peptide (A) and insulin (B) area under the curve in 37 people with normal renal function. Regression line Spearman's r_s correlations shown. *=p<0.0001

In patients with CKD, UCPCR does not correlate with serum C-peptide, insulin or HOMA2-IR

In patients with CKD, median fasting (1.2 v 0.7 nmol/L p<0.0001) and stimulated (457 v 294 nmol/L, p<0.0001)serum C-peptide measures were higher than the subjects without CKD, but serum insulin levels were not different (7685 v 6180 , p=0.4). Despite the higher level of serum C-peptide UCPCR0 was not different between the two groups(1.0 v 0.8, p=0.8) and UCPCR120 was lower in the CKD group (3.8 v 2.7, p=0.02). This is consistent with reduced renal clearance of C-peptide.

For peer review only - http://bmjopen.bmj.com/site/about/guidelines.xhtml

Figure 3

Figure 3: Scatter plots showing no association in patients with CKD between (A) fasting C-peptide and second void UCPCR (r_s 0.17, p=0.4), and (B) fasting insulin and second void UCPCR (r_s -0.17, p=0.4)

In contrast to healthy controls, there was no correlation between UCPCR0 and fasting serum C-peptide (r_s 0.17, p=0.4), insulin (r_s -0.17, p=0.4) or HOMA-IR(r_s -0.16, p=0.4), and no correlation between UCPCR120 and C-peptide (r_s=-0.09, p=1) or insulin area under the curve during the OGTT (r_s=0.26, p=0.2).(Figure 3)

DISCUSSION

Our study suggests that a fasting 2nd void morning UCPCR could be used as a marker of insulin resistance in subjects without diabetes, as long as they are known not to have chronic renal disease. The fact that this test can be done at home without the assistance of healthcare or research staff offers the opportunity to perform a simple assessment of insulin resistance in large scale epidemiological studies.

UCPCR is not a replacement for established measures of insulin resistance, but is an alternative measure of fasting insulin. Numerous population based studies have used HOMA to estimate insulin resistance [9]. Similarly, there are many studies using euglycaemic clamps, and alternative methods such as minimal model analysis to study individual patients or small groups of patients. UCPCR cannot be used as a direct substitute for these as it only measures C-peptide, and although it shows a strong correlation with HOMA2-IR, we have not validated it against the euglycaemic-hyperglycaemic clamp. The similarity of the scatter plots for UCPCR0 against HOMA2-IR and UCPCR0 against fasting insulin demonstrates the large effect that fasting insulin values have on HOMA-IR when subjects do not have abnormal fasting glucose. If fasting glucose levels are elevated, UCPCR will not correlate so well with HOMA-IR as elevated glucose will start to have an effect on the calculation. This suggests that UCPCR may only be useful as a marker of insulin resistance in populations who have

For peer review only - http://bmjopen.bmj.com/site/about/guidelines.xhtml

For peer review only

normal glucose tolerance. There was a correlation between HOMA2-IR, age and BMI in our study, but the variance explained by these simple measure was less than UCPCR0 (r^2=0.27 for BMI v r^2=0.48 for UCPCR0), suggesting it has additional benefit over these measures. UCPCR is a non-invasive test and does not need proximity to a laboratory for immediate sample analysis. Rather than replacing more complex measures of assessment of insulin secretion or resistance, UCPCR is an alternative where serum insulin or C-peptide analysis are impractical, or the non-invasive nature of a urine test is preferred.

We collected second void fasting urine samples because we have shown this to be less variable than first void urine in people without diabetes[12]. This is because C-peptide secretion in response to the previous evening's meal will accumulate in an overnight urine sample. A second void sample adds an extra methodological step which may make sampling more difficult in large studies. It would be interesting to see how well first void urine correlated with serum insulin and C-peptide and there may be existing studies that have both serum and fasting first void urine samples available to easily test this.

A key finding of this study is that UCPCR0 and UCPCR120 were not correlated with serum C-peptide or insulin in subjects with CKD. When comparing the CKD group to the control subjects, serum C-peptide AUC was elevated in subjects with CKD whereas UCPCR120 was lower. This is explained by the reduced renal clearance of C-peptide in CKD[11], leading to higher C-peptide AUC values and lower UCPCR120 values. This impaired clearance may then explain the lack of correlation in subjects with CKD. The numbers of patients in this study were too small to compare patients with different levels of GFR, underlying causes of CKD and the presence of proteinuria. Further work will be needed to fully understand the clearance of C-peptide in people with CKD. These data suggest that UCPCR should not be used in people without diabetes who have CKD. In our previously published study on patients with T2D, mild CKD (in 23 subjects) did not alter the association between UCPCR and serum C-peptide[15]. It is possible that the presence of diabetes, more severe CKD (median eGFR 33(27-46) v 51(44-58) in Bowman's study), or relatively small numbers in both studies may explain the difference between these two sets of results. Our results suggest that further work may be needed to assess the utility of UCPCR in subjects with diabetes and renal impairment.

For peer review only - http://bmjopen.bmj.com/site/about/guidelines.xhtml

This study is important because of the simplicity and practicality of a UCPCR test rather than an ability to more accurately describe insulin physiology in individual subjects. Current measures of insulin secretion and sensitivity rely on serum assays of C-peptide and insulin which require access to centrifugation and freezing within 24 hours. This limits studies to centres with these facilities and staff to use them. UCPCR could be particularly useful in the developing world where the diagnosis of diabetes is rising fastest and reduced facility and staffing costs associated with a posted urine sample may make large studies easier to do. Given the results with CKD and the effect of elevated glucose levels on HOMA calculated insulin resistance, UCPCR may be most useful in young or middle aged populations where the background prevalence of CKD and diabetes is low.

In conclusion UCPCR0 and UCPCR120 correlate with serum levels of insulin and C-peptide, and also with HOMA2 calculated insulin resistance in patients without diabetes. The practical aspects of performing UCPCR testing make it a potentially useful method for the assessment of insulin production and resistance in large epidemiology studies. Patients with CKD should be excluded from these studies.

ACKNOWLEDGEMENTS

Material costs for the project were provided by a Royal Devon and Exeter NHS Foundation Trust Small Projects Grant. ATH, BAK and BMS are core members of the NIHR Exeter Clinical Research Facility and ATH is an NIHR Senior Research Fellow. This study was supported by PenCLAHRC. RAO is funded by Diabetes UK Clinical Training Fellowship.

For peer review only - http://bmjopen.bmj.com/site/about/guidelines.xhtml

REFERENCES

1. Martin BC, Warram JH, Krolewski AS, Bergman RN, Soeldner JS, Kahn CR. Role of glucose and insulin resistance in development of type 2 diabetes mellitus: results of a 25-year follow-up study. *Lancet* 1992;340(8825):925-9.
2. Bonora E, Kiechl S, Willeit J, Oberhollenzer F, Egger G, Meigs JB, et al. Insulin resistance as estimated by homeostasis model assessment predicts incident symptomatic cardiovascular disease in caucasian subjects from the general population: the Bruneck study. *Diabetes care* 2007;30(2):318-24.
3. Polonsky KS, Given BD, Hirsch L, Shapiro ET, Tillil H, Beebe C, et al. Quantitative study of insulin secretion and clearance in normal and obese subjects. *The Journal of clinical investigation* 1988;81(2):435-41.
4. DeFronzo RA, Tobin JD, Andres R. Glucose clamp technique: a method for quantifying insulin secretion and resistance. *Am. J. Physiol.* 1979;237:E214-23.
5. Ferrari P, Alleman Y, Shaw S, Riesen W, Weidmann P. Reproducibility of insulin sensitivity measured by the minimal model method. *Diabetologia* 1991;34(7):527-30.
6. Haffner SM, Miettinen H, Stern MP. The homeostasis model in the San Antonio Heart Study. *Diabetes care* 1997;20(7):1087-92.
7. Stumvoll M, Mitrakou A, Pimenta W, Jenssen T, Yki-Jarvinen H, Van Haeften T, et al. Use of the oral glucose tolerance test to assess insulin release and insulin sensitivity. *Diabetes care* 2000;23(3):295-301.
8. Matthews DR, Hosker JP, Rudenski AS, Naylor BA, Treacher DF, Turner RC. Homeostasis model assessment: insulin resistance and beta-cell function from fasting plasma glucose and insulin concentrations in man. *Diabetologia* 1985;28(7):412-9.
9. Wallace TM, Levy JC, Matthews DR. Use and abuse of HOMA modeling. *Diabetes care* 2004;27(6):1487-95.
10. McDonald TJ, Perry MH, Peake RW, Pullan NJ, O'Connor J, Shields BM, et al. EDTA improves stability of whole blood C-peptide and insulin to over 24 hours at room temperature. *PLoS One* 2012;7(7):e42084.
11. Horwitz DL, Rubenstein AH, Katz AI. Quantitation of human pancreatic beta-cell function by immunoassay of C-peptide in urine. *Diabetes* 1977;26(1):30-5.
12. McDonald TJ, Knight BA, Shields BM, Bowman P, Salzmann MB, Hattersley AT. Stability and reproducibility of a single-sample urinary C-peptide/creatinine ratio and its correlation with 24-h urinary C-peptide. *Clinical chemistry* 2009;55(11):2035-9.
13. Besser RE, Ludvigsson J, Jones AG, McDonald TJ, Shields BM, Knight BA, et al. Urine C-peptide creatinine ratio is a noninvasive alternative to the mixed-meal tolerance test in children and adults with type 1 diabetes. *Diabetes care* 2011;34(3):607-9.
14. Jones AG, Besser RE, McDonald TJ, Shields BM, Hope SV, Bowman P, et al. Urine C-peptide creatinine ratio is an alternative to stimulated serum C-peptide measurement in late-onset, insulin-treated diabetes. *Diabetic medicine : a journal of the British Diabetic Association* 2011;28(9):1034-8.
15. Bowman P, McDonald TJ, Shields BM, Knight BA, Hattersley AT. Validation of a single-sample urinary C-peptide creatinine ratio as a reproducible alternative to serum C-peptide in patients with Type 2 diabetes. *Diabet Med* 2012;29(1):90-3.
16. Organisation WH. Definition and Diagnosis of Diabetes mellitus and intermediate hyperglycaemia. 2006.

For peer review only - http://bmjopen.bmj.com/site/about/guidelines.xhtml

17. Organisation WH. Use of Glycated Haemoglobin (HbA1c) in the diagnosis of Diabetes Mellitus, Abbreviated report of a WHO consultation. 2011.
18. Levey AS, Bosch JP, Lewis JB, Greene T, Rogers N, Roth D. A more accurate method to estimate glomerular filtration rate from serum creatinine: a new prediction equation. Modification of Diet in Renal Disease Study Group. *Annals of internal medicine* 1999;130(6):461-70.

For peer review only

For peer review only - http://bmjopen.bmj.com/site/about/guidelines.xhtml

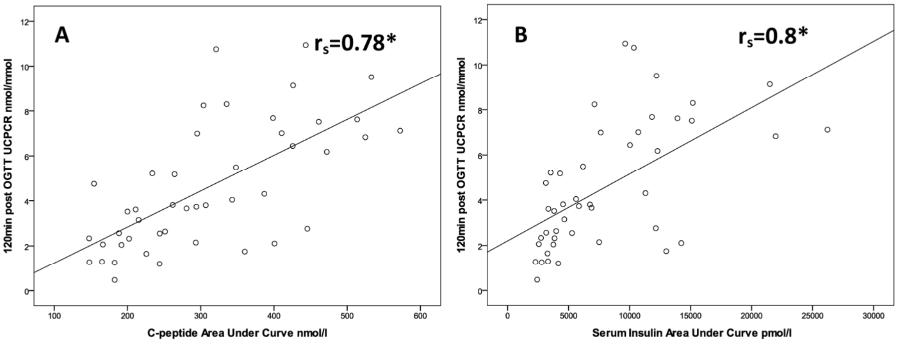

157x58mm (300 x 300 DPI)

For peer review only

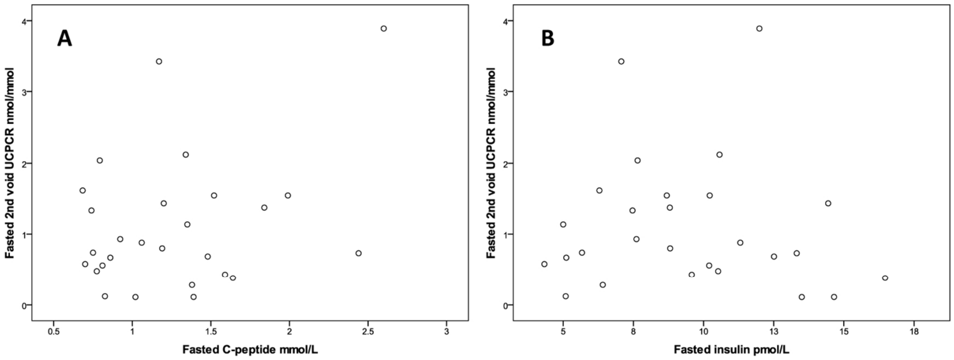

154x60mm (300 x 300 DPI)

For peer review only

STROBE Statement—checklist of items that should be included in reports of observational studies

	Item No	Recommendation
**Title and abstract**	1	Comparison of one technique to another - explained in abstract
**Introduction**		
Background/rationale	2	done
Objectives	3	done
**Methods**		
Study design	4	done
Setting	5	done
Participants	6	done
Variables	7	done
Data sources/ measurement	8*	done
Bias	9	done
Study size	10	As were looking for a correlation rather than a difference between two
Quantitative variables	11	Data were analysed continuously
Statistical methods	12	done

Continued on next page

For peer review only

**Results**		
Participants	13*	done
Descriptive data	14*	done
Outcome data	15*	*Comparison of methods using correlation. Bland Altman not possible as measuring different substance .*
Main results	16	Continuous variables analysed using nonparametric testing due to non normal distribution of data.
Other analyses	17	Analysis of CKD group shown separately
**Discussion**		
Key results	18	done
Limitations	19	done
Interpretation	20	done
Generalisability	21	discussed
**Other information**		
Funding	22	Listed in aknowledgements

*Give information separately for cases and controls in case-control studies and, if applicable, for exposed and unexposed groups in cohort and cross-sectional studies.

Note: An Explanation and Elaboration article discusses each checklist item and gives methodological background and published examples of transparent reporting. The STROBE checklist is best used in conjunction with this article (freely available on the Web sites of PLoS Medicine at http://www.plosmedicine.org/, Annals of Internal Medicine at http://www.annals.org/, and Epidemiology at http://www.epidem.com/). Information on the STROBE Initiative is available at www.strobe-statement.org.

For peer review only

For peer review only - http://bmjopen.bmj.com/site/about/guidelines.xhtml
